# Predictors of recurrence after conversion therapy in unresectable hepatocellular carcinoma treated with HAIC, bevacizumab, and sintilimab

**DOI:** 10.3389/fimmu.2025.1644570

**Published:** 2025-08-26

**Authors:** Chang-Fu Liu, Xiao-Hui Zhao, Shi-Bo Zhu, Hai-Peng Yu, Wen-Ge Xing, Hui-Kai Li

**Affiliations:** ^1^ Department of Interventional Therapy, Tianjin Medical University Cancer Institute & Hospital, National Clinical Research Center for Cancer, Tianjin’s Clinical Research Center for Cancer, Tianjin Key Laboratory of Digestive Cancer, Tianjin, China; ^2^ Department of Hepatobiliary, Tianjin Medical University Cancer Institute & Hospital, National Clinical Research Center for Cancer, Tianjin’s Clinical Research Center for Cancer, Tianjin Key Laboratory of Digestive Cancer, Tianjin, China

**Keywords:** hepatocellular carcinoma, conversion therapy, hepatic arterial infusion chemotherapy, bevacizumab, sintilimab, recurrence

## Abstract

**Background:**

Conversion therapy with hepatic arterial infusion chemotherapy (HAIC) combined with bevacizumab and sintilimab has shown promise for unresectable hepatocellular carcinoma (uHCC). However, predictors of postoperative recurrence remain unclear.

**Methods:**

We retrospectively analyzed 112 HCC patients treated with HAIC + bevacizumab + sintilimab followed by surgical resection. Patients were stratified into recurrence (n = 30) and non-recurrence (n = 82) groups. Demographics, laboratory values, and tumor measurements were collected before and after conversion therapy. Recurrence-free survival (RFS) was estimated by Kaplan–Meier analysis. Restricted cubic spline (RCS) logistic regression was used to identify thresholds for AFP decline and tumor size decline associated with 1-year recurrence. Multivariable logistic regression was used to determine independent predictors of recurrence.

**Results:**

During conversion therapy, the non-recurrence group exhibited greater tumor shrinkage (5.67 ± 3.06 cm vs. 8.77 ± 3.92 cm; p<0.001), lower ALT (p=0.017), higher AST (p=0.008), and lower bilirubin (p=0.006). The median RFS was 22.2 months (95% CI: 18.3–28.0); the 1- and 2-year RFS rates were 71.7% and 46.9%, respectively. The RCS model showed that an AFP decline greater than 25% and tumor size reduction significantly lowered the risk of 1-year recurrence, but reductions in tumor size beyond 60% did not confer additional benefits in reducing recurrence risk. In multivariate analysis, tumor size decline ratio (OR=0.002; 95% CI: 0.000–0.117; p=0.002) and AFP decline ratio (OR=0.240; 95% CI: 0.067–0.862; p=0.029) during conversion therapy independently predicted a lower recurrence risk. Elevated post-therapy bilirubin level remained an adverse predictor (OR=1.020; 95%CI: 1.000–1.030; p=0.039). Adverse events were predominantly grade 1–2, and grade 3–4 adverse events were manageable and well-controlled.

**Conclusions:**

Decline ratios of tumor size and AFP during HAIC + bevacizumab + sintilimab conversion therapy were robust and independent predictors of 1-year postoperative recurrence in HCC. Monitoring of these dynamic biomarkers may guide optimal surgical timing and follow-up strategies.

## Introduction

Traditional treatment modalities have failed to achieve long-term survival in the majority of patients with unresectable hepatocellular carcinoma (uHCC) (1–3). With the continuous development of tyrosine kinase inhibitors (TKIs), immune checkpoint inhibitors (ICIs), and local therapies, conversion therapy has gradually emerged as a novel comprehensive treatment strategy (4, 5). Conversion therapy aims to transform an initially unresectable liver cancer into a resectable state through the combined use of local and systemic treatments, thus providing patients with the opportunity for curative resection and improving long-term survival.

Among these approaches, the combination of TKIs and ICIs has shown promising efficacy in conversion therapy, with conversion success rates ranging from 12.8% to 42.4% (6, 7). Bevacizumab, an anti-angiogenic agent, combined with sintilimab, a PD-1 inhibitor, has emerged as a potential therapeutic strategy for unresectable hepatocellular carcinoma by synergistically targeting the tumor vasculature and enhancing anti-tumor immunity (8). As a critical local treatment, hepatic arterial infusion chemotherapy (HAIC) effectively reduces tumor volume and burden, while improving liver function, creating favorable conditions for subsequent surgical resection through localized high-concentration drug infusion and systemic immune modulation (9).

Despite the hope that conversion therapy is offered to patients with uHCC, postoperative recurrence remains a significant challenge following conversion surgery (10, 11). Clinically, a subset of patients experience tumor recurrence within a short period (e.g., within 3 months) after conversion surgery, which severely affects the long-term survival and overall treatment efficacy. Early recurrence not only suggests limited antitumor effects of conversion therapy in some patients, but also reflects the heterogeneity in tumor biology, immune status, and liver function among individuals (12, 13). Existing studies on conversion therapy for uHCC primarily focus on conversion success rates, surgical safety, and overall survival, with limited systematic analyses of risk factors for early postoperative recurrence (6, 7, 14). Therefore, we conducted a retrospective cohort study based on HAIC combined with TKIs and ICIs for the treatment of uHCC to investigate the postoperative recurrence after conversion surgery. By comparing baseline data at initial diagnosis with preoperative data, this study aimed to explore the association between dynamic changes in clinical indicators and early recurrence. This will help to elucidate the differential efficacy of conversion therapy in different patients and provide a theoretical basis for optimizing treatment protocols and reducing early recurrence rates.

## Materials and methods

### Study design and patient selection

This was a single-center retrospective cohort study. Clinical data were collected from patients who underwent HAIC combined with TKIs and ICIs as conversion therapy, followed by successful surgical resection in the interventional therapy and hepatobiliary oncology departments between November 2019 and December 2023. The inclusion criteria were as follows: (1) diagnosis of hepatocellular carcinoma (HCC) confirmed by pathological or clinical criteria according to the Chinese Guidelines for the Diagnosis and Treatment of Primary Liver Cancer (2019 Edition). (2) All patients received HAIC combined with TKIs and ICIs as the initial conversion therapy at diagnosis and ultimately underwent successful conversion followed by surgical resection. (3) Postoperative follow-up of at least 3 months with comprehensive documentation of the recurrence status. (4) Complete baseline clinical data (at diagnosis and preoperatively) and treatment-related parameters during therapy. Exclusion criteria: (1) Concurrent malignancies or severe systemic diseases significantly affecting prognosis. (2) Major alterations or interruptions in conversion therapy regimens that compromise data integrity. (3) Missing or incomplete follow-up documentation.

All patients were evaluated by a hepatobiliary tumor multidisciplinary team (MDT). “Unresectable” HCC was defined as (1) surgical unresectability, inability to tolerate surgery due to poor systemic condition, insufficient future liver remnant (FLR), or compromised liver function. (2) Oncological unresectability: Technically resectable tumors with no survival benefit compared to non-surgical therapies.

This study used anonymized data from electronic medical records and imaging archives. The study protocol was approved by the Institutional Review Board (IRB) and adhered to the Declaration of Helsinki and data privacy regulations.

### Treatment schedule

The patients received bevacizumab (7.5 mg/kg, intravenous [IV] infusion) and sintilimab (200 mg, IV infusion) on day 1 of each 21-day cycle. Hepatic arterial infusion chemotherapy (HAIC) with the FOLFOX regimen (oxaliplatin 85 mg/m², leucovorin 400 mg/m², fluorouracil bolus 400 mg/m², followed by fluorouracil 2400 mg/m² continuous infusion over 46 h) was administered on day 15 of the same cycle. Treatment was continued for a maximum of six cycles or until disease progression or intolerable toxicity occurred. The treatment regimen typically comprised 4–6 cycles, with radiological (e.g., contrast-enhanced MRI) and hematological assessments guiding therapeutic response monitoring until surgical eligibility criteria were met. Upon meeting the conversion criteria, patients underwent either partial hepatectomy or hepatic tumor resection. The surgical approach was determined by an experienced multidisciplinary hepatobiliary team based on preoperative imaging and clinical status, with intraoperative documentation of resection margins and surgical complications.

### Adjuvant therapy after conversion surgery

Patients who underwent curative resection received two additional cycles of adjuvant therapy with bevacizumab (7.5 mg/kg, intravenous [IV] every 3 weeks) and sintilimab (200 mg, IV every 3 weeks), initiated within 4–6 weeks postoperatively based on recovery status. Each cycle spanned 21 days, with treatment discontinued upon completion of 2 cycles, disease recurrence, or unacceptable toxicity. Dose adjustments (e.g., bevacizumab withholding for grade ≥3 hypertension/proteinuria, sintilimab discontinuation for immune-related adverse events ≥grade 3) and treatment discontinuation followed predefined protocols aligned with CTCAE v5.0.

### Follow-up strategy and assessment methods

The follow-up strategy in this study was adjusted dynamically according to the treatment phase. During the conversion (neoadjuvant) therapy period, patients were followed up at the end of each treatment cycle every 2–4 weeks, with a focus on monitoring treatment response and safety. In the first month after surgery, patients underwent intensive follow-up (once per month) to enable the early detection of surgery-related complications such as hemorrhage, infection, and hepatic decompensation. Thereafter, they entered the long-term follow-up phase: from 2 to 24 months postoperatively, follow-up visits occurred every 3 months, and after 24 months, the interval was extended to every 6 months based on the patient’s clinical status. All patients were followed up for at least 3 months after surgery.

A multimodal assessment system was employed during the follow-up. In terms of imaging, enhanced CT or MRI of the upper abdomen (multiphase) was performed 3 weeks after every two treatment cycles, strictly following the mRECIST criteria to evaluate changes in the sum of the longest diameters of target lesions and to identify any new lesions. Tumor markers included AFP (threshold >20 ng/mL) and PIVKA-II as primary indicators. For suspected progression, CA19–9 and CEA were added. A continuous increase of >50% in two consecutive measurements was considered an alert signal. Organ function assessments included liver function (ALT, AST, TBil, Alb, and PT-INR), renal function (Scr and eGFR), and immunotherapy-related endocrine function (thyroid hormone, cortisol, and ACTH). The overall status was evaluated using the ECOG performance score, body weight trends, and adverse events documented according to the CTCAE v5.0.

### Study endpoints

The primary endpoint of this study was early postoperative recurrence, defined as radiologically confirmed tumor recurrence within 12 months of hepatectomy. Confirmation required meeting the following criteria: (1) Enhanced CT or MRI showing a newly developed lesion with arterial-phase hyperenhancement and washout in the portal venous phase; (2) lesion measuring ≥1 cm in diameter, confirmed by at least two imaging techniques; and (3) exclusion of postoperative inflammatory pseudotumors, vascular malformations, and other non-neoplastic lesions.

Secondary endpoints included recurrence-free survival (RFS) and the incidence of Clavien-Dindo grade III or higher surgical complications.

To ensure the accuracy of endpoint assessments, a multi-level quality control system was established. (1) All radiologic data were independently reviewed in a blinded manner by two senior radiologists, and any discrepancies were referred to a multidisciplinary team for discussion. (2) For patients with a marked increase in AFP/PIVKA-II but negative imaging results, PET-CT or hepatic angiography was performed. (3) If a suspicious lesion was identified, ultrasound-guided biopsy was used to obtain histological confirmation, when feasible.

### Statistical analysis

All statistical analyses were performed using SPSS software (version 16) or R language. Continuous variables were expressed as mean ± standard deviation or median (interquartile range), while categorical variables were presented as frequencies and percentages. To compare baseline characteristics between the recurrence and non-recurrence groups, independent-sample t-tests, Mann–Whitney U tests, chi-square tests, or Fisher’s exact tests were employed as appropriate. Restricted cubic spline (RCS) analysis was used to evaluate the relationship between continuous variables and outcomes. Variables that achieved statistical significance in the univariate analyses were subsequently entered into a multivariate logistic regression model. Additionally, both univariate and multivariate Cox regression analyses were conducted to identify prognostic factors affecting recurrence-free survival (RFS). Statistical significance was defined as a two-sided P-value <0.05.

## Results

### Patient characteristics

A total of 106 patients were included in this study and divided into recurrence and non-recurrence groups based on tumor relapse. The baseline characteristics of the two groups were comparable. The median age was 57.37 ± 9.74 years in the non-recurrence group and 59.23 ± 9.37 years in the recurrence group (p = 0.372). Although the recurrence group had a higher proportion of males (96.7% vs. 81.6%), this difference was not statistically significant (p = 0.089). The ECOG performance status was similar between the groups (p = 1.000), and hepatitis B status was comparable, with 26.3% and 26.7% of patients testing positive in the non-recurrence and recurrence groups, respectively (p = 1.000). Regarding tumor characteristics, the distribution of CNCL stage showed a trend toward differences (p = 0.076); stage IIa disease was observed in 25.0% of the non-recurrence group compared to 50.0% in the recurrence group, while the distribution of other stages was similar. Child-Pugh scores did not differ significantly (p = 0.715), and the proportions of patients with solitary versus multiple tumors were also comparable between the groups (p = 0.622). The mean number of HAIC cycles was 3.72 ± 1.33 in the non-recurrence group and 3.50 ± 1.36 in the recurrence group (p = 0.441).

Before conversion therapy, there were no significant differences between the groups in tumor size (9.51 ± 4.13 cm vs. 10.60 ± 3.89 cm, p = 0.216), baseline AFP levels, CA 19-9, albumin, ALT, AST, bilirubin, hemoglobin, white blood cell count, or platelet count (all p > 0.05). However, significant differences were observed after conversion therapy. The non-recurrence group exhibited a significantly smaller tumor size (5.67 ± 3.06 cm) than the recurrence group (8.77 ± 3.92 cm, p < 0.001). Although post-conversion AFP levels did not differ significantly (p = 0.536), other biochemical markers did differ. Specifically, the non-recurrence group had significantly lower ALT values (45.88 ± 35.76 U/L vs. 70.13 ± 66.89 U/L, p = 0.017) and significantly higher AST values (36.74 ± 6.34 U/L vs. 33.17 ± U/L, p = 0.008). Moreover, the bilirubin level was significantly lower in the non-recurrence group (17.07 ± 10.88 µmol/L) compared to the recurrence group (28.22 ± 30.08 µmol/L, p = 0.006). There were no significant differences in post-treatment hemoglobin, white blood cell, or platelet counts between the groups.

All patients received two cycles of adjuvant therapy with bevacizumab and sintilimab. Among them, 9 patients discontinued therapy early due to grade ≥3 adverse events, including immune-related hepatitis (n = 2), hypertension (n = 4), and fatigue (n = 3). In exploratory analysis, there was no statistically significant difference in 1-year recurrence between patients who completed therapy and those who discontinued adjuvant therapy (p>0.05), although the sample size was limited.

### Survival analysis

**Figure 1:**
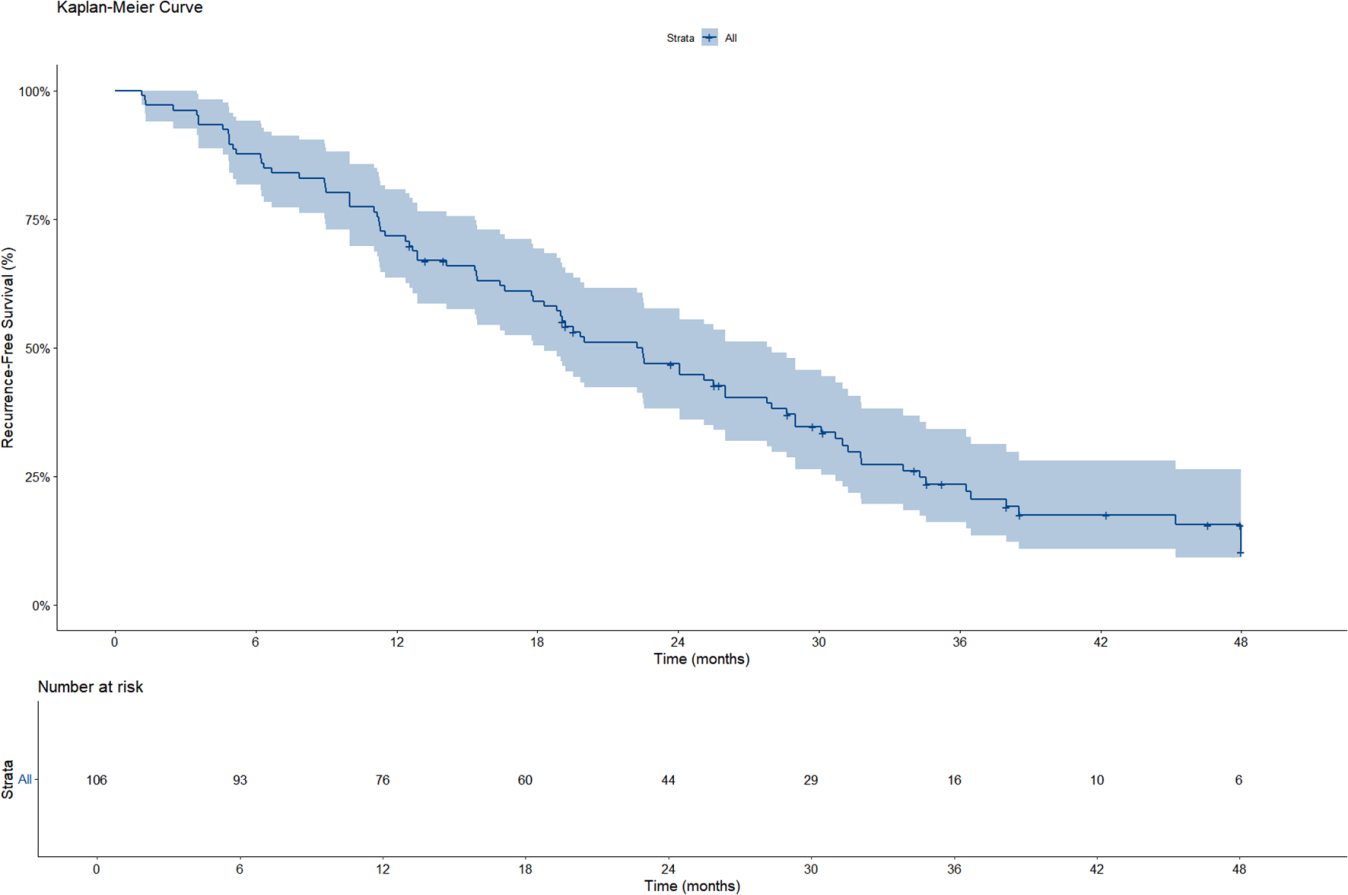
KM curve of RFS.

A total of 106 patients were included in the RFS analysis, of whom 82 experienced disease recurrence during follow-up. As shown in the Kaplan–Meier curve, the median RFS was 22.2 months (95%CI: 18.3–28.0) (**Figure 1**). Based on the Kaplan–Meier analysis, the estimated 1−year RFS rate was 71.7% and the 2−year RFS rate was 46.9%.

Among the 30 patients who experienced recurrence within 1 year, 20 cases (66.7%) were classified as intrahepatic recurrence, 5 cases (16.7%) as extrahepatic recurrence (including lung and bone metastases), and 5 cases (16.7%) as combined intra- and extrahepatic recurrence.

### Association of decline ratios with 1-year recurrence

**Figure 2:**
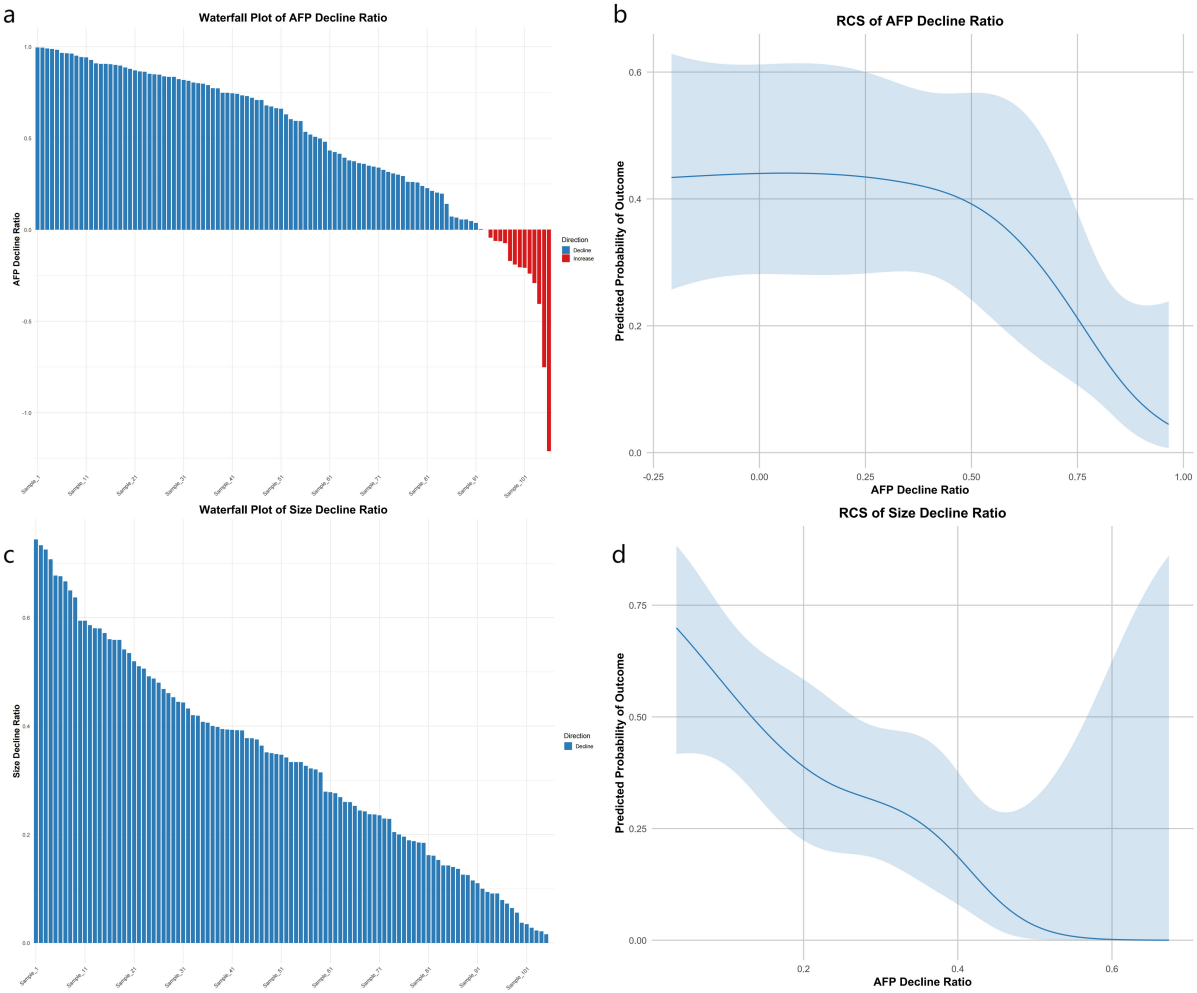
Four-panel plot linking AFP and tumor size decline to 1-year recurrence. **(a)** Waterfall of AFP decline ratios: blue bars for decline, red for increase, ordered by magnitude. **(b)** RCS logistic curve of AFP decline vs. predicted recurrence risk; shaded 95% CI shows risk drop after ~25% decline. **(c)** Waterfall of size decline ratios (blue bars). **(d)** RCS curve of size decline vs. recurrence risk; CI shading shows risk plateau beyond ~60% reduction. X-axes: decline ratios; Y-axes: ratio **(a, c)** or predicted probability **(b, d)**.

Using restricted cubic spline logistic regression, we investigated the association between the decline rates of AFP (Model 1), tumor size (Model 2), and 1-year recurrence (**Figure 2**). Following Harrell’s recommendation, we utilized four knots and performed piecewise regression, such that each segment met the linearity assumption. The final results indicated that both models were essentially linear, with Model 1 yielding a chi-square value of 13.9 (p = 0.002) and Model 2 a chi-square of 30.28 (p < 0.001). The waterfall plot for the AFP decline ratio revealed that most patients achieved varying degrees of AFP reduction (**Figure 2a**), although a subset of patients experienced an increase in AFP. The AFP decline model demonstrated a significant inverse relationship with recurrence risk: as the AFP decline rate increased, the probability of recurrence decreased. However, this relationship was not uniform across the entire range; when the decline ratio was negative (indicating an increase in AFP), the recurrence probability appeared to be higher; in the 0–25% decline range, the recurrence probability remained relatively unchanged; and when the decline ratio exceeded 25%, the recurrence probability significantly dropped (**Figure 2b**). Similarly, the waterfall plot for the tumor size reduction ratio showed that all patients experienced some degree of tumor size reduction (**Figure 2c**). The tumor size decline model also exhibited a predominantly inverse and nonlinear relationship with 1-year recurrence, where larger reductions in tumor size were generally associated with a lower predicted recurrence risk, reaching a plateau at a reduction of approximately 60% (**Figure 2d**).

### Subgroup RCS modeling for recurrence risk stratification

**Figure 3:**
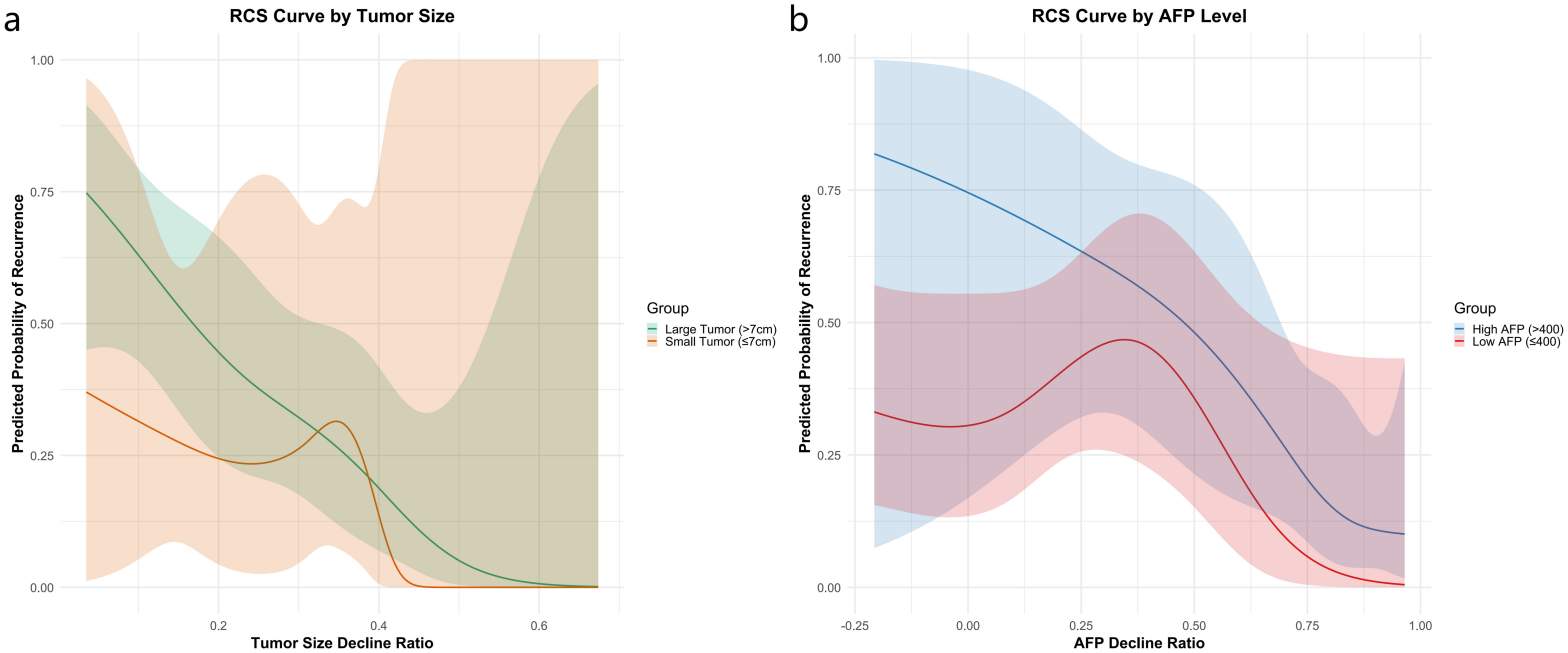
Recurrence risk stratification using RCS analysis by baseline AFP level **(a)** and tumor size **(b)**.

To further assess the prognostic value of dynamic changes in AFP and tumor size, we performed stratified RCS analyses according to baseline AFP levels and baseline tumor size. For AFP stratification, patients were divided into two subgroups: high-AFP (baseline AFP >400 ng/mL) and low-AFP (≤400 ng/mL). As shown in **Figure 3a**, the RCS curves revealed that patients in the high-AFP group exhibited consistently higher predicted recurrence probabilities across most of the AFP decline range. In contrast, the low-AFP group demonstrated a non-linear trend, with a transient rise in recurrence probability around a 25% AFP decline, followed by a gradual decrease. This fluctuation may reflect the limited prognostic utility of AFP dynamics in patients with initially low AFP levels.

For tumor size stratification, patients were grouped using a 7 cm baseline cutoff. As illustrated in **Figure 3b**, patients with larger tumors (>7 cm) showed substantially higher recurrence risk, particularly when tumor shrinkage was less than 32%. Among patients with smaller tumors (≤7 cm), modest size reductions had limited effect on recurrence risk; however, a sharp risk reduction was observed when tumor shrinkage exceeded approximately 35%, indicating that significant tumor regression is especially beneficial in this subgroup.

### Multivariable analysis of factors associated with 1-year recurrence

Multivariable analysis was performed by incorporating the variables that showed differences in **Table 1**, along with the decline rates of tumor size (Size_ decline_ratio) and AFP (AFP_ decline_ratio), to evaluate the factors associated with 1−year recurrence. During the conversion therapy phase, the tumor size decline rate was significantly associated with recurrence; specifically, the size _ decline_ratio showed an odds ratio (OR) of 0.002 (95% CI: 0.000–0.117, P = 0.002), indicating that greater tumor shrinkage conferred a marked protective effect. Similarly, a higher AFP decline rate was significantly associated with a lower recurrence risk, with an OR of 0.240 (95% CI: 0.067–0.862, P = 0.029). In contrast, after conversion therapy, none of the tested parameters was significantly associated with 1−year recurrence: tumor size (OR = 1.062, 95% CI: 0.896–1.260, P = 0.488), AST (OR = 1.008, 95% CI: 0.994–1.022, P = 0.273), albumin (OR = 0.976, 95% CI: 0.871–1.094, P = 0.679), and WBC count (OR = 1.025, 95% CI: 0.989–1.062, P = 0.182) (**Table 2**). These findings suggest that the decline in tumor size and AFP level prior to conversion therapy are important predictors of early recurrence, whereas the post-conversion parameters do not independently influence recurrence risk.

**Table 1:** Baseline characteristics of the patients.

Variables	n (%)		P
	Non-recurrence group	Recurrent group	
Age (years)	57.37 ± 9.74	59.23 ± 9.37	0.372
Sex			
Male	62 (81.6%)	29 (96.7%)	0.089
Female	14 (18.4%)	1 (3.3%)	
ECOGPS			
0	62 (81.6%)	24 (80.0%)	1.000
1	14 (18.4%)	6 (20.0%)	
Hepatitis B			
Yes	20 (26.3%)	8 (26.7%)	1.000
No	56 (73.7%)	22 (73.3%)	
CNCL stage			
Ib	15 (19.7%)	4 (13.3%)	0.076
IIa	19 (25.0%)	15 (50.0%)	
IIb	33 (43.4%)	10 (33.3%)	
IIIa	9 (11.8%)	1 (3.3%)	
Child-Pugh score			
A	60 (78.9%)	22 (73.3%)	0.715
B	16 (21.1%)	8 (26.7%)	
HAIC cycles	3.72 (1.33%)	3.50 (1.36%)	0.441
Tumor number (/)			
multiple	15 (19.7%)	4 (13.3%)	0.622
solitary	61 (80.3%)	26 (86.7%)	
Before conversion therapy			
Tumor size (cm)	9.51 ± 4.13	10.60 ± 3.89	0.216
AFP (ng/ml)			
<400	16	37	0.667
≥400	14	39	
CA19-9 (U/ml)	89.85 ± 355.32	41.55 ± 45.47	0.461
ALB (g/l)	44.92 ± 39.41	67.83 ± 112.56	0.122
ALT (U/L)	63.13 ± 42.17	100.53 ± 196.11	0.116
AST (U/L)	39.51 ± 9.43	37.55 ± 6.13	0.295
BIL (µmol/L)	21.48 ± 17.99	18.37 ± 11.14	0.379
Hb (g/L)	135.62 ± 20.73	136.60 ± 20.18	0.825
WBC (10^9/L)	5.62 ± 2.46	5.96 ± 2.51	0.533
PLT (10^9/L)	168.41 ± 80.66	183.87 ± 74.49	0.366
After conversion therapy			
Tumor size (cm)	5.67 ± 3.06	8.77 ± 3.92	<0.001
AFP (ng/ml)			
<400	17	48	0.536
≥400	13	28	
CA19-9 (U/ml)	34.37 ± 26.47	44.94 ± 77.75	0.297
ALB (g/l)	34.46 ± 30.20	40.07 ± 29.64	0.389
ALT (U/L)	45.88 ± 35.76	70.13 ± 66.89	0.017
AST (U/L)	36.74 ± 6.34	33.17 ± 5.42	0.008
BIL (µmol/L)	17.07 ± 10.88	28.22 ± 30.08	0.006
Hb (g/L)	128.83 ± 21.46	124.90 ± 23.08	0.408
WBC (10^9/L)	5.36 ± 2.78	6.17 ± 3.23	0.197
PLT (10^9/L)	131.97 ± 81.11	153.40 ± 91.14	0.240

**Table 2:** Multivariate analysis of factors affecting recurrence.

Variables	Multivariate analysis	
	OR (95%CI)	P value
Size decline ratio	0.002 (: 0.000–0.117)	0.002
AFP decline ratio	0.240 (: 0.067–0.862	0.029
After conversion therapy		
Tumor size (cm)	1.062 (: 0.896–1.260)	0.488
AST	1.008 (: 0.994–1.022)	0.273
ALB (g/l)	0.976 (: 0.871–1.094)	0.679
BIL	1.025 (: 0.989–1.062)	0.182

### Independent prognostic factors for recurrence and recurrence-free survival

During the conversion therapy phase, the tumor size decline rate was a strong independent predictor of 1-year recurrence. Specifically, Size_decline_ratio exhibited an odds ratio (OR) of 0.070 (95% CI: 0.020–0.270, P = 0.001), indicating that greater tumor shrinkage before conversion therapy was associated with a markedly reduced risk of recurrence. Although the AFP decline ratio showed a slight association with reduced recurrence risk in the univariate analysis (OR = 0.614; 95% CI: 0.370–1.020; P = 0.059), it was not retained in the final multivariate model. Among the remaining baseline variables, the Child-Pugh score was statistically significant in the univariate analysis (OR = 2.059; 95% CI: 1.240–3.421; P = 0.005), but was not significant in the multivariate model (OR = 1.410; 95% CI: 0.780–2.560; P = 0.259).

Univariate analysis of post–conversion therapy parameters revealed that tumor size was significantly associated with recurrence (OR = 1.125; 95% CI: 1.058–1.197; P < 0.001); however, in multivariate analysis, tumor size was not an independent predictor (OR = 1.030; 95% CI: 0.960–1.100; P = 0.451). Among the liver function markers assessed after conversion therapy, bilirubin remained independently associated with recurrence, with an OR of 1.020 (95% CI: 1.000–1.030, P = 0.039), suggesting that higher post-therapy bilirubin levels were modestly linked to an increased risk of recurrence. Other post-therapy parameters, including albumin level (OR, 0.976; 95% CI, 0.871–1.094; P = 0.679), were not statistically significant (**Table 3**).

**Table 3:** Univariate and multivariate analyses of factors affecting RFS.

Variables	Univariate analysis		Multivariate analysis	
	OR (95%CI)	P value	OR (95%CI)	P value
Age	1.007 (0.985-1.030)	0.527		
Sex	0.810 (0.446-1.470)	0.488		
ECOG	1.323 (0.774-2.260)	0.306		
HBV	0.960 (0.596-1.547)	0.867		
CNLC	1.567 (0.804-3.057)	0.187		
	1.735 (0.908-3.315)	0.095		
	2.211 (0.929-5.265)	0.073		
Child-Pugh score	2.059 (1.240-3.421)	**0.005**	1.410 (0.780–2.560)	0.259
HAIC cycles	1.045 (0.889-1.229)	0.592		
Tumor number	1.390 (0.780-2.476)	0.264		
Size decline ratio	0.041 (0.012-0.142)	**<0.001**	0.070 (0.020–0.270)	**0.001**
AFP decline ratio	0.614 (0.370-1.020)	0.059		
Before conversion therapy				
Tumor size (cm)	1.012 (0.959-1.067)	0.672		
AFP (ng/ml)	1.000 (0.999-1.001)	0.331		
CA19-9 (U/ml)	1.000 (0.999-1.001)	0.806		
ALB (g/l)	0.977 (0.948-1.006)	0.118		
ALT	1.002 (0.999-1.005)	0.088		
AST	1.002 (0.999-1.003)	0.062		
BIL	0.999 (0.986-1.012)	0.854		
Hb	0.996 (0.986-1.006)	0.424		
WBC	0.971 (0.888-1.062)	0.517		
PLT	0.998 (0.995-1.001)	0.268		
After conversion therapy				
Tumor size (cm)	1.125 (1.058-1.197)	**<0.001**	1.030 (0.960–1.100)	0.451
AFP (ng/ml)	1.000 (0.999-1.001)	0.908		
CA19-9 (U/ml)	1.004 (0.998-1.010)	0.162		
ALB (g/l)	0.956 (0.917-0.997)	**0.036**	1.010 (0.960–1.060)	0.781
ALT	0.996 (0.989-1.004)	0.358		
AST	1.002 (0.998-1.006)	0.434		
BIL	1.022 (1.009-1.035)	**0.001**	1.020 (1.000–1.030)	**0.039**
Hb	0.993 (0.983-1.004)	0.204		
WBC	1.033 (0.952-1.121)	0.439		
PLT	0.999 (0.996-1.002)	0.471		

The bolded numbers indicate p-values <0.05, demonstrating statistical significance.

### Adverse events

**Figure 4:**
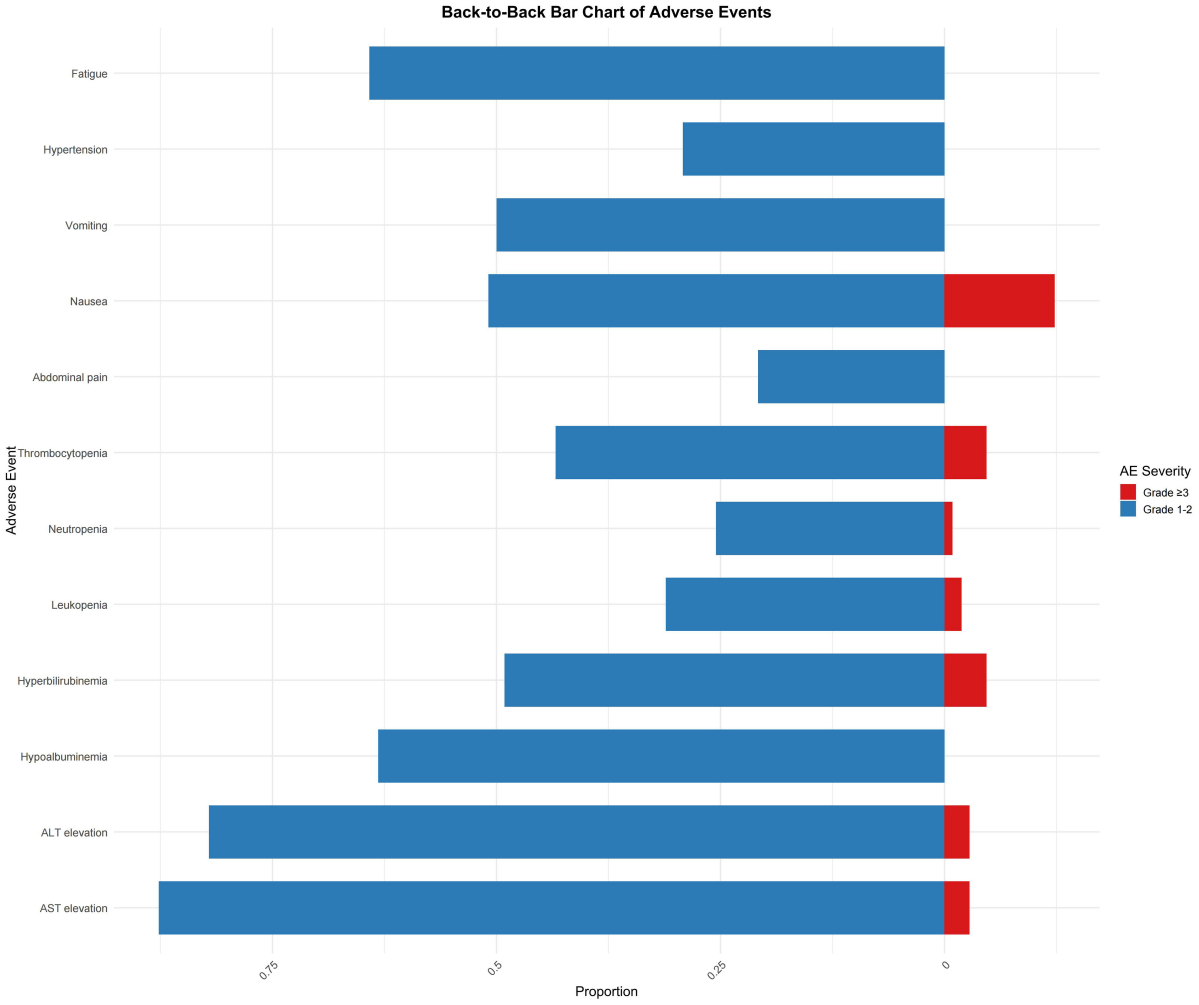
Back-to-back horizontal bar chart of adverse events by severity.

Among the 106 evaluable patients, the most common adverse events (AEs) of any grade included elevated aspartate aminotransferase (AST) and alanine aminotransferase (ALT) levels. AST elevation was observed in 93 patients, with 87.7% occurring as grades 1–2 and 2.8% as grade ≥3. Similarly, ALT elevation was noted in 87 patients, with 82.1% of cases being grade 1–2 and 2.8% being grade ≥3. Hypoalbuminemia was reported in 67 patients (63.2% with grade 1–2 only), whereas hyperbilirubinemia was observed in 52 patients (49.1% with grade 1–2 and 4.7% with grade ≥3). In addition, thrombocytopenia was recorded in 46 patients (43.4% in grade 1–2 and 4.7% in grade ≥3) and leukopenia in 33 patients (31.1% for grade 1–2 and 1.9% for grade ≥3). Among gastrointestinal events, abdominal pain occurred in 54 patients (50.9% for grade 1–2 and 12.3% for grade ≥3), nausea was reported in 53 patients (50.0% for grade 1–2, with no grade ≥3 events), and vomiting occurred in 31 patients (29.2% for grade 1–2 only). Anorexia was observed in 68 patients (64.2%), and diarrhea in 21 patients (19.8% for grade 1–2, and 0.9% for grade ≥3). Additionally, hypertension was reported in 32 patients (30.2% for grades 1–2 and 11.3% for grade ≥3). Less common AEs included hand-foot syndrome (2.8%), oral mucositis (5.7%), gum bleeding (6.6%), and upper gastrointestinal bleeding (3.8% for grades 1–2 and 0.9% for grade ≥3). Finally, fatigue was noted in 65 patients (61.3% at grades 1–2 and 2.8% at grade ≥3) (**Figure 4**).

### Representative case

**Figure 5:**
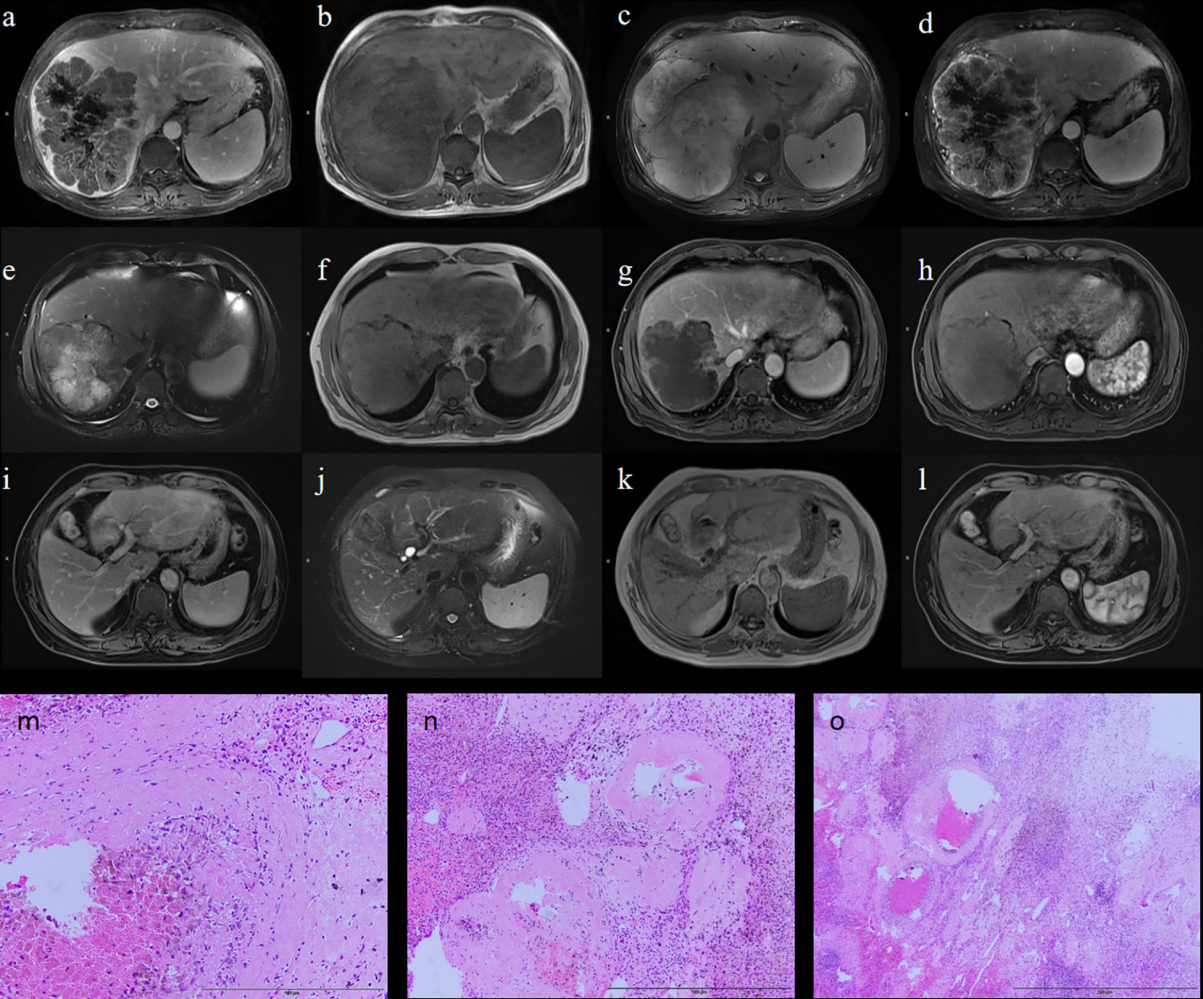
Representative case. Preoperative MRI **(a–d)**; Postoperative MRI **(e–h)**; MRI follow-up at 24 months after treatment **(i–l)**; Intraoperative pathology **(m–o)**.

A 68-year-old male presented with a large, irregular right-lobe hepatic mass measuring 15.6 × 12.8 × 16.2 cm on MRI, with filling defects in the right hepatic vein and inferior vena cava (**Figures 5a–d**). His baseline AFP was 1,143 ng/mL. He received three cycles of HAIC combined with bevacizumab and sintilimab. Post-treatment MRI demonstrated tumor shrinkage to 11.7 × 9.8 × 11.5 cm and decreased enhancement (**Figures 5e–h**); AFP decreased to 649 ng/mL. Given this favorable radiologic and serologic response, he proceeded to curative resection. Pathology revealed extensive necrosis with inflammatory infiltrates and cholesterol crystals, consistent with treatment effect; margins were negative (R0) (**Figures 5m–o**), and 0/2 lymph nodes were involved. Immunohistochemistry showed Arginase-1 (+), Hepatocyte (+), GS (focal +), and a Ki-67 index 2%. At 24-month follow-up, imaging showed postoperative changes without enhancement, and AFP remained within normal limits, indicating no recurrence (**Figures 5i–l**).

## Discussion

This study investigated the predictors of postoperative recurrence in patients with HCC after conversion therapy with HAIC combined with bevacizumab and sintilimab. The results indicated a significant negative correlation between the tumor size reduction rate and AFP decline ratio during conversion therapy, and the risk of recurrence within one year after surgery. Specifically, a greater reduction in tumor size was associated with a lower recurrence risk; however, when the reduction exceeded 60%, the improvement in recurrence risk became less pronounced, possibly because of the increased difficulty of further tumor shrinkage. Similarly, an increase in the AFP decline ratio was correlated with a reduced risk of recurrence, but the recurrence risk only showed a significant decrease when the AFP decline exceeded 25%. From the perspective of unresectable HCC, the tumor size reduction rate and AFP decline ratio, to some extent, reflect the technical and oncological response to unresectability. This highlights the importance of identifying the optimal timing for conversion resection to achieve better prognosis.

Although HAIC combined with bevacizumab and sintilimab improves tumor resectability, postoperative recurrence remains a critical issue affecting long-term survival. This study found that common biomarkers, such as tumor size, AST, and albumin after conversion therapy, were not independently associated with recurrence, suggesting that the mechanisms of postoperative recurrence are complex. Single clinical or biological markers may not be sufficient to predict recurrence risk. Notably, this study also found that patients with higher bilirubin levels postoperatively had a higher risk of recurrence, highlighting the potential role of liver function in postoperative recurrence (15, 16).

In previous studies, HAIC combined with bevacizumab and sintilimab as conversion therapy has demonstrated promising efficacy in patients with uHCC. For example, Liu et al. reported that 19 of 29 patients (65.5%) were eligible for further treatment, of whom 14 successfully underwent surgical resection (14). As a local chemotherapy strategy, HAIC enhances antitumor effects by directly delivering chemotherapeutic agents to the liver tumor site, thereby increasing the local drug concentration. This combination therapy leverages the synergistic effects of localized chemotherapy and systemic immune modulation, resulting in improved patient responses and higher conversion success rates (17). For instance, Zhang et al. demonstrated that HAIC combined with lenvatinib and PD-1 inhibitors significantly increased the objective response rate (31.25%) in patients with advanced hepatocellular carcinoma with macrovascular invasion (18). Other studies have also shown that HAIC in combination with TKIs and ICIs effectively delays disease progression and improves patient survival outcomes (19, 20). Despite these encouraging findings, postoperative recurrence remains a major challenge that affects long-term survival.

In the study by Deng et al., HAIC alone was used as the primary conversion therapy, with a median RFS of 14.1 months (95% CI, 11.4–16.8 months). The 1-year and 2-year RFS rates were 57.3% and 42.9%, respectively. In contrast, the median RFS in our study was 22.2 months (95% CI, 18.3–28.0), which was significantly longer than that observed in the study by Deng et al. This difference could be attributed to the additional effects of bevacizumab and sintilimab. However, there were notable differences in baseline characteristics between the two studies; specifically, only 11.8% of patients in our study were in stage IIIa compared with 37.4% in Deng et al. Patients with stage IIIa HCC, which is often associated with vascular invasion, are known to have a higher risk of postoperative recurrence (21).

Regarding prognostic factors for recurrence, previous studies have identified tumor number, AFP response, tumor response, and successful downstaging as independent predictors of RFS (13, 22). Building on prior findings, our study further explored the impact of tumor size reduction and AFP decline ratio on postoperative recurrence during conversion therapy. We found that substantial reductions in tumor size and AFP levels were significantly correlated with a lower risk of recurrence one year postoperatively. Additionally, we identified two critical thresholds: 60% tumor size reduction and 25% AFP decline. Stratified RCS analyses further revealed that the prognostic value of these dynamic changes varied by baseline characteristics. In patients with high baseline AFP (>400 ng/mL), recurrence risk remained elevated despite AFP decline, whereas in those with low AFP (≤400 ng/mL), the relationship was non-linear, suggesting limited predictive value. Likewise, tumor shrinkage had a greater impact in patients with smaller baseline tumors (≤7 cm), where a reduction beyond 35% led to a sharp drop in recurrence risk. These findings emphasize the importance of personalized risk assessment based on baseline AFP and tumor burden to guide conversion therapy and postoperative surveillance strategies. Although HAIC combined with bevacizumab and sintilimab has been shown to enhance the preoperative conversion rate in patients with uHCC (14), predictive factors for postoperative recurrence, particularly dynamic changes across different treatment stages (such as preoperative and postoperative periods), have not been thoroughly investigated.

On the other hand, although adjuvant therapy was administered uniformly, its potential impact on recurrence remains uncertain. A small subset of patients discontinued treatment due to toxicity, yet recurrence outcomes did not differ significantly between those who completed versus those who discontinued therapy. However, because of the limited sample size, definitive conclusions cannot be drawn. Further randomized studies are warranted to determine whether adjuvant immunotherapy meaningfully reduces recurrence risk.

Furthermore, our study found that elevated bilirubin levels were strongly associated with an increased risk of postoperative recurrence, which may be linked to liver dysfunction. This suggests that the liver function status may play a critical role in the risk of recurrence after surgery (23–25). Additionally, the albumin-bilirubin (ALBI) grade, a recently proposed biomarker for assessing liver reserve before surgery, has emerged as a potential surrogate predictor of tumor recurrence after resection. Although not the primary focus of our study, this observation highlights the potential utility of ALBI classification in predicting postoperative outcomes.

This study has some limitations. First, as bevacizumab and sintilimab have only been widely applied in clinical practice in recent years, the follow-up period in our study was relatively short, and we did not have sufficient follow-up data to evaluate OS outcomes. Second, to avoid variations across different TKIs and ICIs, we included only bevacizumab and sintilimab as systemic therapy options, which undoubtedly limits the generalizability of our findings to other regimens. However, the primary focus of our study was to explore the impact of key indicator changes on outcomes with the aim of identifying the optimal timing for conversion therapy. Third, although all included patients successfully underwent conversion surgery, this was a single-center retrospective study and selection bias may exist. In particular, conversion success criteria may vary from center to center. At our center, conversion failure was defined as failure to meet surgical criteria due to disease progression, poor liver function, or insufficient tumor shrinkage after 4–6 cycles of treatment. Finally, as this was a single-center study, the generalizability of our findings may be limited, underscoring the need for large-scale, multicenter studies to validate our results.

Overall, this study provides new insights into the prediction of postoperative recurrence and underscores the importance of tumor size reduction and AFP level decline as dynamic indicators. Nevertheless, the prediction of postoperative recurrence should be integrated with more clinical and molecular markers to achieve more accurate risk assessment and support personalized treatment strategies.

## Data Availability

The raw data supporting the conclusions of this article will be made available by the authors, without undue reservation.
